# Efficacy of silicone sheet as a personalized barrier for preventing adhesion reformation after hysteroscopic adhesiolysis of intrauterine adhesions

**DOI:** 10.1002/rmb2.12294

**Published:** 2019-09-06

**Authors:** Atsushi Azumaguchi, Hirofumi Henmi, Tsuyoshi Saito

**Affiliations:** ^1^ Sapporo Endometrium Research Sapporo Japan; ^2^ Department of Gynecology and Reproductive Endocrinology, National Public Service Mutual Aid Association Tonan Hospital Sapporo Japan; ^3^ Department of Obstetrics and Gynecology Sapporo Medical University Sapporo Japan

**Keywords:** hysteroscopic adhesiolysis, intrauterine adhesions, intrauterine devices, oxidized regenerated cellulose, silicones

## Abstract

**Purpose:**

To evaluate the efficacy of silicone sheet as a new type of barrier for preventing adhesion reformation following hysteroscopic adhesiolysis of intrauterine adhesions (IUAs).

**Methods:**

Hysteroscopic adhesiolysis was performed for 36 patients with IUAs. The adhesion reformation rate was retrospectively compared between 26 patients treated with silicone sheet (group 1) and 10 patients treated with an intrauterine device wrapped in oxidized regenerated cellulose as a barrier (group 2). For patients in group 1, a 1‐mm‐thick silicone sheet was cut to fit the size and shape of the individual uterine cavity as a personalized barrier.

**Results:**

The size and shape of each silicone sheet used for patients in group 1 differed significantly. The adhesion reformation rate was significantly lower in group 1 (4/26, 15.4%) than in group 2 (4/10, 40.0%; *P* = 0.03), although the pregnancy rate (14/20, 70.0% vs. 5/10, 50.0%; *P* = 0.28) and miscarriage rate (2/14, 14.3% vs. 1/5, 20.0%; *P* = 0.72) were not significantly different.

**Conclusion:**

Use of silicone sheets appears to be effective for preventing adhesion reformation following hysteroscopic adhesiolysis of IUAs. This is the first study to investigate the efficacy of silicone sheet used as a personalized barrier for preventing IUAs.

## 1 INTRODUCTION

Intrauterine adhesions (IUAs) are predominately a consequence of endometrial injuries that cause partial or complete obstruction within the uterine cavity and/or cervical canal, resulting in menstrual disorders and infertility. IUAs can be treated by hysteroscopic adhesiolysis, but adhesion recurrence following surgery is not uncommon. The prevention of adhesion reformation using a barrier between facing endometrial wounds thus becomes necessary in most cases. A wide range of adhesion reformation rates following hysteroscopic adhesiolysis has been reported depending on the barrier used and the patients’ characteristics.^1^, ^2^, ^3^, ^4^, ^5^, ^6^, ^7^, ^8^, ^9^, ^10^

Contraceptive intrauterine devices (IUDs), balloons, and hyaluronic acid gel have all been used as barriers. However, neither IUDs nor Foley catheters were specifically developed as barriers for preventing IUAs, and many devices may be unsuitable for preventing marginal wall adhesions due to their shape. Furthermore, use of a Foley catheter has been shown to increase pressure on endometrial tissue, which may interfere with regeneration and even cause necrosis.^11^ Consequently, a Foley catheter has not been inserted in the uterus for more than 10 days.^12^ The Cook balloon uterine stent was developed as a barrier for preventing IUAs.^13^ However, the Cook balloon uterine stent should not be inserted for more than a week, because prolonged placement may increase the risk of intrauterine infection.^14^ Regardless of the type of device used, the size and shape of these devices (ie, IUD, Foley catheter, or Cook balloon uterine stent) cannot be altered to fit uterine cavities of differing shapes and sizes. These devices are therefore unsuitable for use in patients with uterine cavities that have become enlarged or deformed due to adenomyosis, myoma, or an arcuate uterus. Hyaluronic acid gel can act as a barrier even in an enlarged or deformed uterine cavity, but it has not been shown to be effective for a sufficiently prolonged duration.^15^ Auto‐cross‐linked hyaluronic acid gel has not yet been confirmed as effective beyond 72 hours, according to an ultrasonographic investigation.^15^

To resolve the problems associated with these barriers, silicone sheets or IUDs wrapped in oxidized regenerated cellulose (ORC) have been used as a new type of barrier at Tonan Hospital since 2004. The size and shape of silicone sheets are easily modified, and we hypothesized that ORC might be effective for an enlarged or deformed uterine cavity by melting and extending outside the IUD.

## 2 MATERIAL AND METHODS

This retrospective study was approved by the institutional review board of the National Public Service Mutual Aid Association, Tonan Hospital. The adhesion reformation rate, pregnancy rate, and miscarriage rate following hysteroscopic adhesiolysis for IUAs were compared between two groups: group 1, patients treated with silicone sheet (Silicone Sheet; Koken, Tokyo, Japan), originally indicated for covering surgical wounds in the field of plastic surgery; and group 2, patients treated with an IUD (FD‐1; Fuji Latex, Tokyo, Japan) wrapped in ORC (Interceed; Johnson and Johnson, Tokyo, Japan) as a barrier for preventing re‐adhesion. Clinical results were also investigated in the subgroups of patients with mild, moderate, or severe IUA defined by the American Society for Reproductive Medicine.^16^ When adhesion reformation occurred, further operation was recommended and performed for some patients, but only first operations were included in this study, because the efficacy of first and subsequent surgeries cannot be compared.

A total of 39 first hysteroscopic adhesiolysis procedures were performed for IUA between November 2004 and August 2014 at Tonan Hospital. Three patients were excluded from this study: one who did not attend the hospital for postoperative removal of the silicone sheet, preventing confirmation of results; one whose operation contained two different procedures (adhesiolysis and septectomy); and one whose silicone sheet was expelled from the uterine cavity 8 days after surgery, followed soon after by placement of an IUD wrapped in ORC, so that which procedure or barrier impacted the result could not be determined. Thus, 36 patients were enrolled in this study. Of these 36 patients, 26 were treated by a silicone sheet and 10 by an IUD wrapped in ORC. All operations were conducted by the same two authors (A.A and H.H). Although the selection of barrier to be used was made by the surgeon, the IUD wrapped in ORC tended to be chosen for patients with milder adhesions.

**Figure 1:**
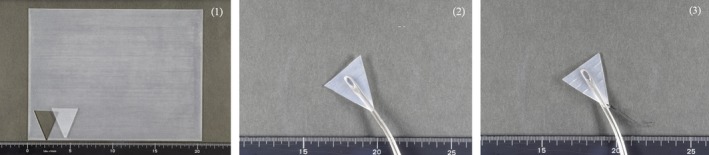
Referring to findings of hysterosalpingography, a 200 × 150 × 1 mm^3^ silicone sheet is cut to fit the size and shape of the intrauterine cavity, then sterilized and preserved until surgery (1). Following adhesiolysis, the silicone sheet is inserted into the cavity using small placental forceps, and the fit with the cavity is observed using hysteroscopy. When necessary, the sheet can be pulled out and the size or shape corrected as many times as needed (2). After confirming an appropriate fit, the silicone sheet with slits and thread is placed in the cavity (3)

**Figure 2:**
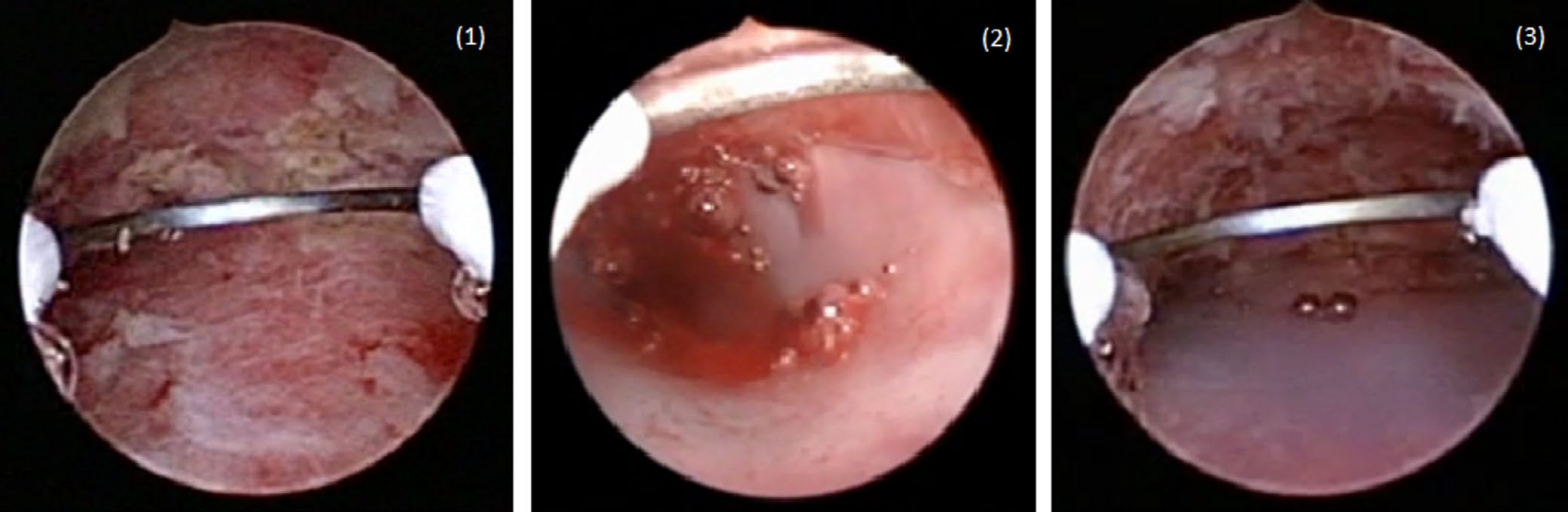
Uterine cavity where adhesiolysis was accomplished (1). Silicone sheet is bent and inappropriately placed in the uterine cavity (2). Silicone sheet is flat and appropriately placed in the uterine cavity (3)

**Figure 3:**
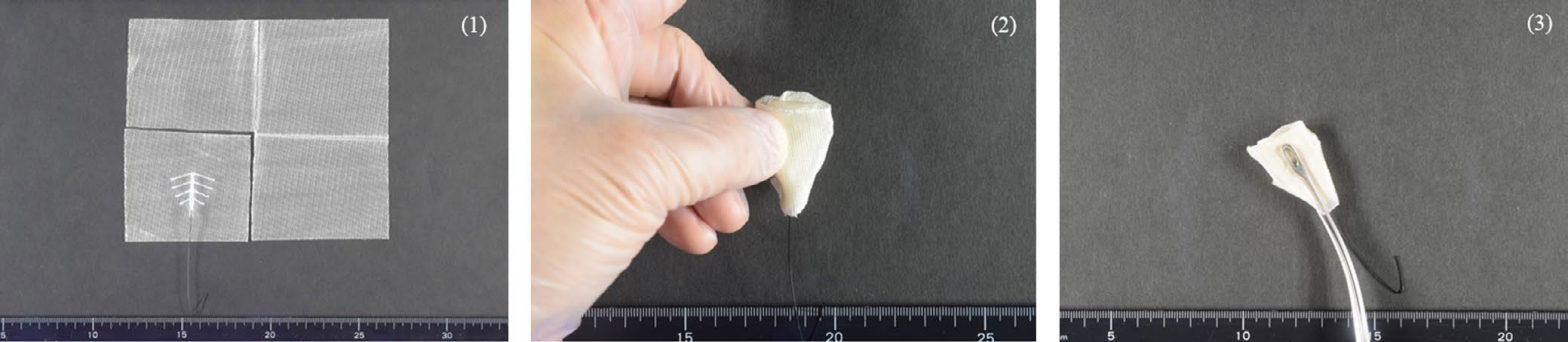
The intrauterine device is manually wrapped in oxidized regenerated cellulose (1/4 of the original 12.7 × 15.2 cm^2^ size) (1,2) and placed in the uterine cavity using small placental forceps (3)

For patients in group 1, referring to the findings of size and shape observed by hysterosalpingography, a silicone sheet (200 × 150 × 1 mm^3^) was cut to fit the size and shape of the uterine cavity, then sterilized and preserved until surgery. When adhesion was identified in the cervical canal, the lower part of the silicone sheet was made long and wide. Following adhesiolysis during surgery, the silicone sheet was inserted into the uterine cavity using small placental forceps, and then the fitness of the silicone sheet in the uterine cavity was observed by hysteroscopy. When necessary, the sheet was pulled out, and the size and/or shape corrected as many times as needed. After confirming an appropriate fit, six slits were made in the sheet to prevent the sheet from slipping out of the uterine cavity, nylon thread was threaded through a small hole in the lower part of the sheet to allow easy retraction after insertion, and the device was placed in the uterine cavity (Figures 1 and 2). For patients in group 2, an IUD was manually wrapped in ORC (1/4 of the original 12.7 × 15.2 cm^2^ size) and inserted into the uterine cavity using small placental forceps (Figure 3). For smooth insertion of the IUD wrapped in ORC, the cervical canal was dilated sufficiently. Written, informed consent for use of both the silicone sheet and IUD wrapped in ORC was obtained from all patients. Patients were given conjugated estrogen (1.875 mg/day) for 20 days along with additional dydrogesterone (15 mg/day) for the last 10 days after surgery. Both barriers were removed after the second withdrawal bleeding following surgery at an outpatient clinic. Further hysteroscopy was performed to examine the status of adhesion reformation after removal of the barriers.

JMP version 12 software (SAS Institute, Cary, NC) was used for statistical analysis. Student's *t*‐test was used to compare age between groups. The χ^2^ test was used to compare the frequencies of causes of IUA and infertility. Comparisons of the adhesion reformation rate, pregnancy rate, and miscarriage rate were performed using the Cochran‐Mantel‐Haenszel test, considering the grade of adhesion. Statistical significance was set at *P* < 0.05.

## 3 RESULTS

**Figure 4:**
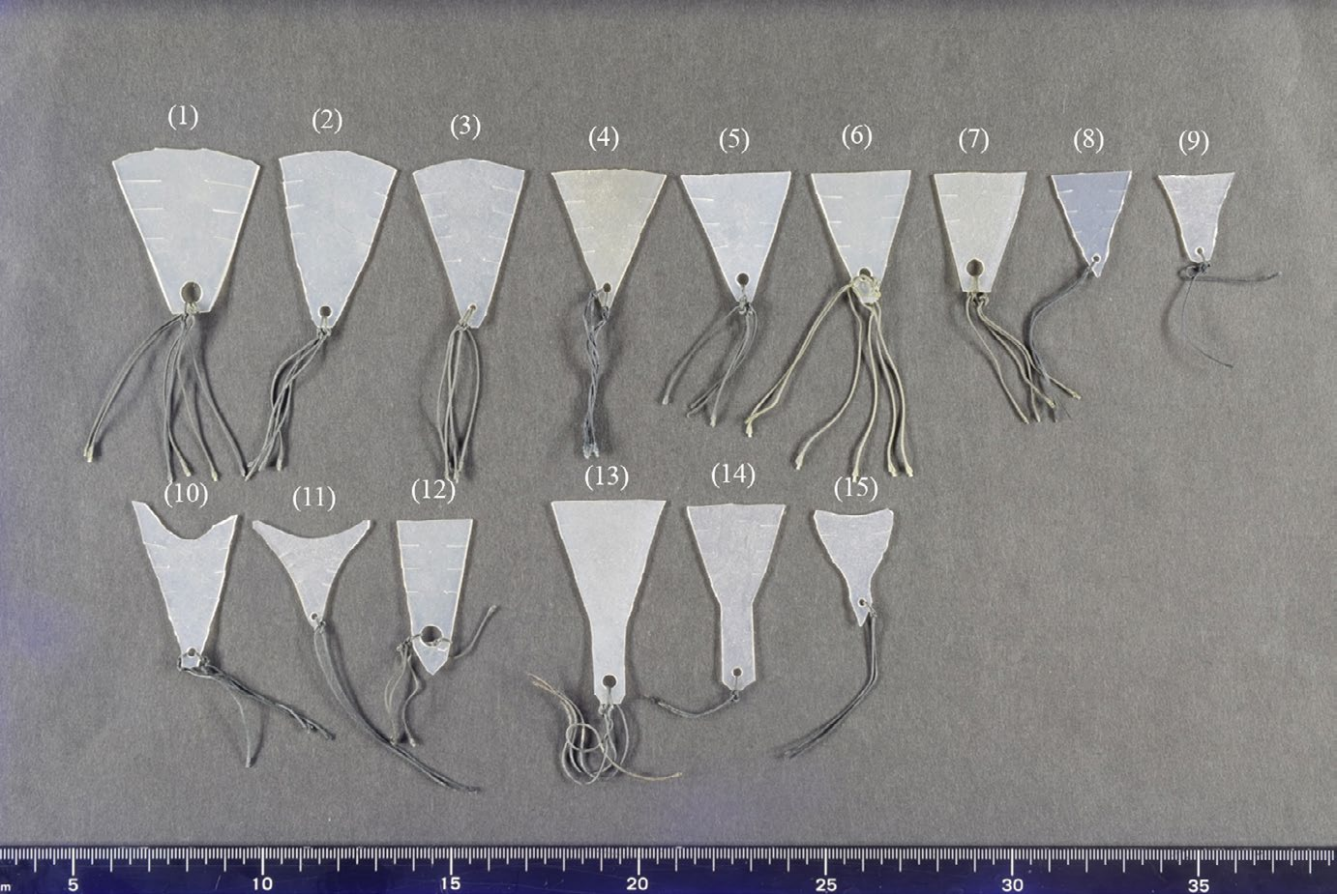
Silicone sheets, with different sizes (1‐9) and deformed shape (10‐12), used in patients. For patients with adhesions in the cervical canal, a silicone sheet with a wide lower part is used (13‐15)

The size and shape of each silicone sheet used for the 26 patients in group 1 differed significantly (Figure 4). Patient age, suspected causes of IUA, and causes of infertility did not differ between the patients of both groups (Table 1). The adhesion reformation rate was significantly lower for group 1 (4/26, 15.4%) than for group 2 (4/10, 40.0%; *P* = 0.03), even though no patients in group 2 showed severe IUA. Thirty of the 36 patients were trying to conceive. The overall pregnancy rate did not differ between group 1 (14/20, 70.0%) and group 2 (5/10, 50.0%; *P* = 0.28). Five of the 14 pregnancies in group 1 and one of 5 pregnancies in group 2 were achieved using assisted reproductive technology, and the others were natural pregnancies. The overall miscarriage rate did not differ significantly between group 1 (2/14, 14.3%) and group 2 (1/5, 20.0%; *P* = 0.72) (Table 2).

**Table 1:** Clinical parameters of patients

	Silicone sheet	IUD + ORC	*P*‐value
Number of patients	26	10	
Age (y)	35.3 ± 4.0	37.4 ± 2.8	0.14
Causes of IUA			0.85
D&C for spontanous abortion	8	7	
D&C for induced abortion	4	1	
D&S for spontaneous abortion	2	1	
D&C for endometrial polyps	1	0	
D&C for endometrial hyperplasia	1	0	
Hysteroscopic myomectomy	2	0	
Abdominal myomectomy	3	1	
Laparoscopic myomectomy	1	0	
Uterine artery embolism	1	0	
Uterine septectomy	1	0	
Cesarian section	1	0	
D&C postpartum	1	0	
Number of infertile patients	20	10	
Causes of infertility			0.68
IUA only	5	5	
Thin endometrium	11	3	
Tubal factor	4	1	
Endometriosis	3	1	
Male factor	2	1	
Ovulation factor	1	0	

Abbreviations: IUD, intrauterine device; ORC, oxidized regenerated cellulose; IUA, intrauterine adhesion; D&C, dilatation and curettage; D&S, dilatation and suction.

**Table 2:** Postsurgical results in mild, moderate, and severe IUA groups

	Silicone sheet	IUD + ORC	*P*‐value
Adhesion reformation rate, n/n (%)			0.03
Mild	1/7 (14.3)	1/7 (28.6)	
Moderate	1/12 (8.3)	3/3 (100.0)	
Severe	2/7 (28.6)	None	
Total	4/26 (15.4)	4/10(40.0)	
Pregnancy rate, n/n (%)			0.28
Mild	3/5 (60.0)	4/7 (57.1)	
Moderate	9/11 (81.8)	1/3 (33.3)	
Severe	2/4 (50.0)	None	
Total	14/20 (70.0)	5/10 (50.0)	
Miscarriage rate, n/n (%)			0.72
Mild	0/3 (0.0)	1/4(25.0)	
Moderate	2/9 (22.2)	0/1 (0.0)	
Severe	0/2 (0.0)	None	
Total	2/14 (14.3)	1/5 (20.0)	

Abbreviations: IUA, intrauterine adhesion; IUD, intrauterine device; ORC, oxidized regenerated cellulose.

There was no patient whose silicone sheet was expelled from the uterine cavity after surgery in group 1. Endometrial infection or uterine perforation was not observed, and none of the patients complained of abdominal pain after surgery or during device removal in both groups.

## 4 DISCUSSION

To date, no reports have been published in which the size and shape of IUDs or balloons were modified for use in different endometrial cavities. The present study showed that the size and shape of a 1‐mm‐thick silicone sheet were easily modified, allowing the sheet to be fit into individual uterine cavities, even when these cavities were enlarged or deformed. To the best of our knowledge, this is the first study to investigate the efficacy of silicone sheets used as personalized barriers to prevent IUA.

The adhesion reformation rate of the IUD wrapped in ORC was 40.0% (4/10) in the present study. There has been only one study in which the efficacy of an IUD wrapped in ORC as a barrier was investigated; the adhesion reformation rate was 78.0% (32/41) in patients treated with an IUD wrapped in ORC after hysteroscopic adhesiolysis of IUAs.^6^ There have not been sufficient studies of IUDs wrapped in ORC, but these findings may indicate that the melting of the ORC wrapping the IUD does not enhance the adhesion protecting effect of the IUD.

On the other hand, adhesion reformation rates were significantly lower in group 1 than in group 2, suggesting that the silicone sheet might be more effective than the IUD wrapped in ORC. In addition, the adhesion reformation rate of 15.4% (4/26) in patients treated with silicone sheets in this study was comparable with the results of the studies reporting the lowest adhesion reformation rates among studies done in that last 10 years (Table 3).^1^, ^3^, ^10^

**Table 3:** Studies in the last 10 y investigating adhesion reformation rates after hysteroscopic adhesiolysis of IUAs

Author	Mild (n)	Moderate (n)	Severe (n)	Total (n)	Barrier	Adhesion reformation (n)	Adhesion reformation rate (%)
Roy et al (2010)^1^	31	40	18	89	T‐shape IUD	12	13.5
Kim et al (2012)^2^	Not classified			16	ACH	7	43.8
	Not classified			19	CH	8	42.1
Liu et al (2014)^3^	13	86	54	153	T‐shape IUD	22	14.4
Lin et al (2015)^4^	None	53	29	82	Heart‐shaped balloon	25	30.5
	None	43	37	80	Heart‐shaped copper IUD	28	35.0
Thubert et al (2015)^5^	12	11	9	32	ACP gel	14	53.8
	24	28	6	58	None	22	53.7
Cai et al (2017)^6^	None	12	23	35	Circular Inert IUD	31	88.6
	None	17	24	41	Circular Inert IUD + Interceed	32	78.0
Gan et al (2017)^7^	Not classified			40	Foley balloon covered by amnion	11	27.5
	Not classified			40	Foley balloon	16	40.0
Peng et al (2017)^8^	None	None	40	40	Foley balloon with amnion graft	19	47.5
	None	None	80	80	Foley balloon	36	45.0
Zhu et al (2018)^9^	None	28	46	74	Foley balloon	26	35.1
	None	44	32	76	Intrauterine suitable balloon	19	25.0
Liu et al (2018)^10^	None	83	93	176	None	33	18.8

Abbreviations: IUA, intrauterine adhesion; IUD, intrauterine device; ACH, alginate carboxymethylcellulose hyaluronic acid; CH: carboxymethylcellulose hyaluronic acid; ACP gel, auto‐cross‐linked hyaluronic acid gel

Although the length of time needed for endometrial restoration after adhesiolysis and the duration required for barrier retention in the adhesiolyzed uterine cavity have not been extensively investigated, it has been reported that it took 2 months for complete endometrial wound healing following hysteroscopic polypectomy in 37 patients when observed by hysteroscopy.^17^ This finding suggested that the duration required for the barrier to prevent adhesion reformation after adhesiolysis was at least 2 months. A Foley catheter or Cook balloon uterine stent cannot be placed for 2 months,^12^, ^14^ and hyaluronic acid gel has not been confirmed to be effective beyond 72 hours,^15^ but a silicone sheet can be retained, as with other IUDs, in the uterine cavity for 2 months without any side effects.

This study showed that the size and shape of silicone sheets could be easily modified, and placement in the uterine cavity for a sufficient period was readily achieved. As a result, a markedly low adhesion reformation rate was achieved using silicone sheets as personalized barriers without any side effects. These findings suggest that a silicone sheet could be promising for preventing adhesion reformation after hysteroscopic adhesiolysis of IUAs.

However, this study had some limitations. First, as a non‐randomized, retrospective study, there may have been selection bias owing to the decision made by the surgeon regarding which barrier to use. Second, although patient age and suspected causes of infertility did not differ between the groups, the sample size was small. Considering these limitations, large‐scale, prospective studies are warranted to confirm the efficacy of silicone sheets for this application.

## DISCLOSURES

*Conflict of interest:* The authors declare no conflict of interest. *Human rights statement and informed consent*: This project was reviewed and approved by the institutional review board of the National Public Service Mutual Aid Association, Tonan Hospital, Sapporo, Japan. Informed consent was obtained from all patients in this study. *Animal studies*: This article does not report any work undertaken with animal participants. *Approval by ethics committee*: This study was approved by the ethics committee of the National Public Service Mutual Aid Association, Tonan Hospital (Registration number: 347).
